# Supplementary material for: Molecular and Genomic Analysis of the Virulence Factors and Potential Transmission of Hybrid Enteropathogenic and Enterotoxigenic Escherichia coli (EPEC/ETEC) Strains Isolated in South Korea
Source: Int J Mol Sci. 2023 Aug 12;24(16):12729. doi: 10.3390/ijms241612729 (PMC10454139; doi:10.3390/ijms241612729)
Supplement: Supplementary file 1 [file ijms-24-12729-s001.zip › Figure S1.docx]

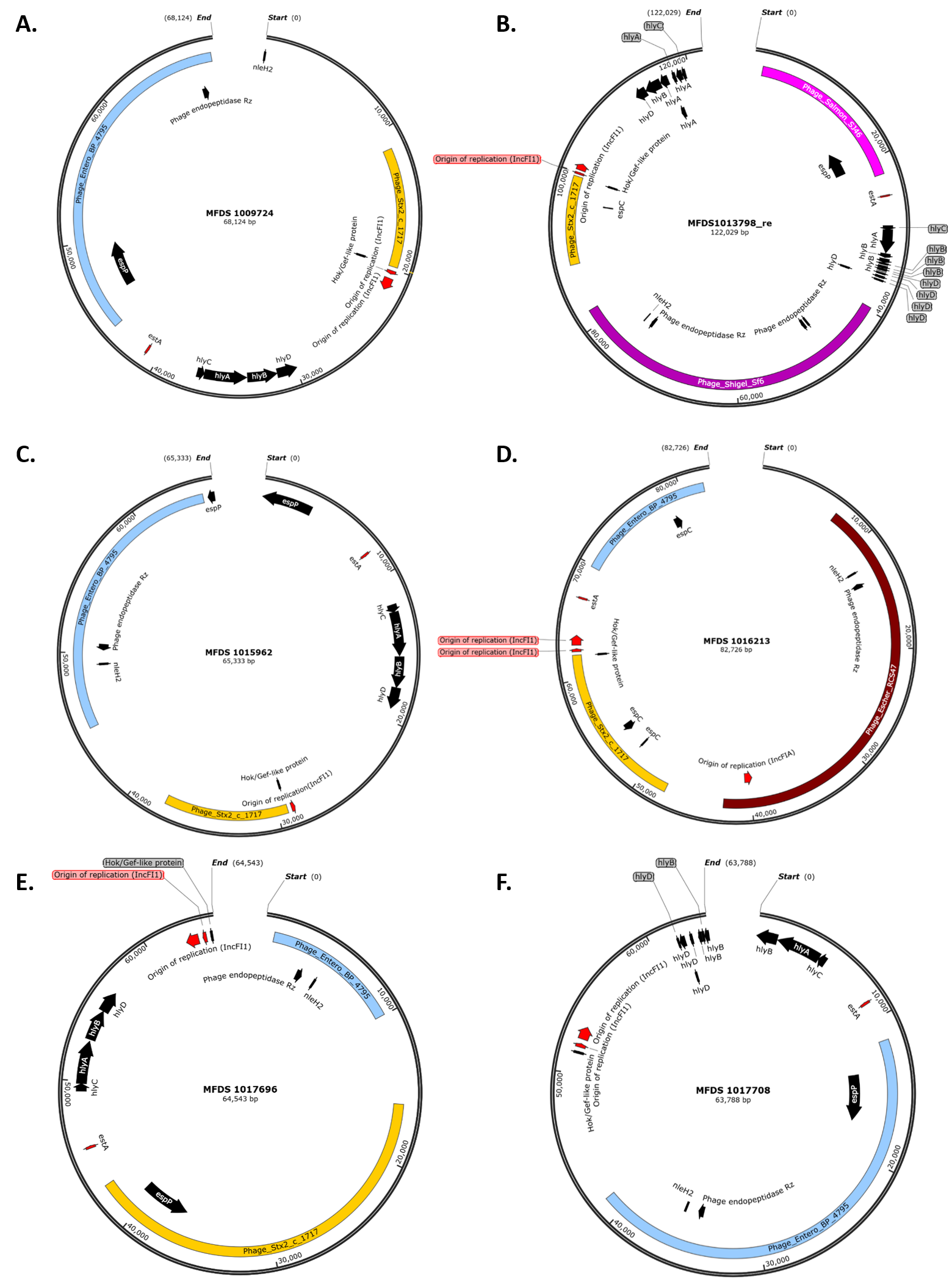


**Figure S1.** Genome map of heat-stable toxins-encoding plasmids. Plasmid replicons, Phages, virulence genes, and transporters are shown. Maps were designed with SnapGene Viewer 6.2.1.
